# Development of an Application Method for Volatile Compounds Derived from Mushroom Fungi Beds as Plant Growth-Promoting Biostimulants

**DOI:** 10.3390/mps8020029

**Published:** 2025-03-07

**Authors:** Clever N. Kanga, Yui Okisaka, Shigeru Hanamata, Daijiro Ueda, Tsutomu Sato, Toshiaki Mitsui, Kimiko Itoh

**Affiliations:** 1 Graduate School of Science and Technology, Niigata University, Niigata 950-2181, Japan; 2 Faculty of Science, Kanagawa University, Yokohama 221-8686, Japan; ss299480bz@jindai.jp; 3 Institute of Science and Technology, Niigata University, Niigata 950-2181, Japan; daijiro@agr.niigata-u.ac.jp (D.U.); satot@agr.niigata-u.ac.jp (T.S.); t.mitsui@agr.niigata-u.ac.jp (T.M.)

**Keywords:** rice, plant growth promotion, non-contact exposure, gas-permeable film, 1-octen-3-ol, 3-octanol, fungal-derived volatiles, HS-SPME-GC-TOF-MS

## Abstract

Volatile compounds (VCs) from fungi can promote plant growth, but their application methods are limited. Edible mushroom fungi beds (FBs) provide a readily available alternative source of fungal VCs, although their biostimulatory functions remain unvalidated. In this study, a novel, non-contact exposure method for applying VCs emitted from FBs to rice seedlings was developed. This marks the first evaluation of mushroom FBs as a direct source of bioactive VCs for plant growth promotion. Volatiles from two different edible mushroom FBs promoted shoot growth and increased biomass for rice seedlings. VCs from shiitake FBs significantly increased biomass by 67.4% while VCs from enokitake FBs by 39.5% compared to the control. The biomass-increasing effects were influenced by the quantity of shiitake FBs applied, with significant increases at 15 g, 30 g and 60 g applications. The VCs effects remained significant even when the FBs were covered with two types of gas-permeable polymer film. Chemical analysis of VCs from FBs identified several organic compounds and subsequent bioassays using synthetic VCs determined key bioactive VCs contributing to biomass increase at specific concentrations. This study presents a utilization method of waste mushroom FBs as sustainable, scalable, and cost-effective agricultural biostimulants.

## 1. Introduction

Volatile compounds (VCs) are low-molecular-weight substances, encompassing both inorganic and organic compounds that diffuse through air, soil, or water, enabling them to interact with organisms and influence biological functions [1,2]. Many organisms emit VCs and interact with each other, and in this study, we focus on the effects of VCs emitted by edible mushroom fungi beds (FBs) on crops. Previous research has consistently demonstrated that fungal VCs act as signaling molecules that promote plant growth, enhance stress tolerance, and improve overall plant health [3,4]

The potential of fungal VCs as plant growth promoters was first highlighted by Ryu et al. (2003), who demonstrated that VCs emitted by fungal mycelia could stimulate plant growth without direct physical contact [5]. Subsequent studies corroborated these findings across diverse fungal and plant species. For instance, Jiang et al. (2021) found that VCs from *Cladosporium halotolerans* NGPF1 significantly improved the growth of horticultural crops, while Rao et al. (2022) reported that *Trichoderma*-derived VCs enhanced tomato seedling growth by promoting root branching and suppressing diseases [6,7]. Similarly, You et al. (2022) demonstrated that *Trichoderma koningiopsis* T-51 VCs improved both the quality of tomato fruits and the biomass of *Arabidopsis thaliana* [8].

A variety of exposure methods have been developed to study the effects of fungal VCs on plant growth. These methods typically involve creating a shared atmosphere between the fungal source and target plants without direct physical contact. Chamber-based co-cultivation systems using Petri dishes, where fungi and plants are grown in separate compartments that share the same headspace, are commonly employed [3]. Additionally, innovative experimental setups such as the modified square Petri dish system introduced by Fiers et al. (2013) [9], the extensively used sandwich methods [10,11,12,13,14,15,16,17], single-plate systems without physical divisions [18,19], split-plate assays [20,21,22,23,24,25], and plate-within-plate configurations used by Lee et al. (2015) to investigate the effect of fungal aging on VOCs production [26] have been explored. While these methods have been crucial for understanding fungal VCs–plant interactions, challenges remain in scaling up their application for agricultural use. Ensuring stability and efficacy under field conditions, optimizing exposure techniques, and identifying low-cost sources of fungal VCs are critical obstacles [27,28,29,30,31].

Despite the demonstrated potential of fungal VCs to promote plant growth, the complexity of cultivating fungi and extracting these VCs from mycelia continues to hinder their widespread practical application. Current approaches often require controlled laboratory conditions, making large-scale implementation challenging. On the other hand, edible mushroom cultivation substrates, also known as FBs, could provide a readily available alternative source of fungal VCs. These FBs, typically made from agricultural by-products such as sawdust or rice straw, are widely available but often discarded as waste after mushroom harvesting. Recent studies suggest that bioactive volatiles released from waste edible mushroom FBs can have protective effects on plants, even after their primary use in mushroom production [32,33]. However, their biostimulatory functions remain largely unvalidated, and no systematic study has assessed their effectiveness as plant growth promoters.

The main aim of this study was to evaluate the potential of mushroom FBs as a direct source of bioactive fungal VCs to promote plant growth. To achieve this, we studied a novel non-contact exposure method for applying the VCs to rice seedlings (*Oryza sativa* L.). The specific objectives of the study were as follows: (i) assess the biostimulatory effects of VCs emitted by two major mushroom species, namely shiitake (*Lentinula edodes*) and enokitake (*Flammulina filiformis*), on the growth and biomass accumulation of rice seedlings; (ii) determine the optimal FBs dose for maximum plant growth by using FBs quantities ranging from 1 g to 60 g; (iii) investigate the effects of encasing FBs in gas-permeable polymer films; (iv) examine the effect of FBs maturity stages on VCs emissions and their effects on plant growth promotion; and (v) identify key bioactive VCs from waste shiitake FBs using Headspace Solid-Phase Microextraction Gas Chromatography Time-of-Flight Mass Spectrometry (HS-SPME-GC-TOF-MS) analysis and to evaluate their individual contributions to plant growth responses.

The methods and protocols presented in this study enable the development of the efficient use of edible mushroom FBs as biostimulants after mushroom production and can be easily applied to the production of quality crop seedlings.

## 2. Experimental Design

### 2.1. FBs Types and Sources

Two mushroom strains and cultivation substrates, referred to as FBs, were obtained from a commercial supplier in Japan (Mori Sangyo Co., Ltd., Kiryu, Japan).

- Shiitake (*Lentinula edodes*): Mori XR1 strain. FBs cultivated under standardized commercial conditions for shiitake mushroom production.
- Enokitake (*Flammulina filiformis*): Mori No.75 strain. FBs cultivated under standardized commercial conditions for shiitake mushroom production.

For studies examining the effects of fungi bed aging, shiitake FBs were further categorized into three stages based on their lifecycle:

- Young shiitake FBs (YSFB)—initial colonization with minimal fruiting;
- Mature shiitake FBs (MSFB)—peak fruiting and harvest stage;
- Waste shiitake FBs (WSFB)—post-harvest substrates considered exhausted but containing residual mycelium.

All mushroom FBs substrates were transported in sealed packaging to maintain sterility and moisture and stored at 4 °C until further use.

### 2.2. Plant Material and Culture Conditions

- Plant material: Rice seeds (*Oryza sativa* L. cv. Nipponbare) were used in this study. Rice seeds used in this study were harvested under controlled greenhouse conditions during autumn 2021.
- Culture soil: Rice seedlings were grown in Granular Kumiai Synthetic Culture Soil No. 3 (TAKARA Industry Co., Ltd., Tokyo, Japan), characterized by consistent nutrient content (N: 1.5 g, P: 2.7 g, K: 1.5 g per 2.7–3.0 kg soil). To normalize the soil pH, we added calcium silicate (JA ZEN-NOH Co., Ltd., Tokyo, Japan) using the standard 1/4 spoon (0.25 cc capacity, 108 mm overall length; Wadasuke Manufacturing Co., Ltd., Niigata, Japan). The soil was sterilized using an autoclave (HICLAVE HVE-50, HIRAYAMA Manufacturing Co., Ltd., Tokyo, Japan) at 121 °C, 0.20 Mpa for 20 min, and then the soil was kept to room temperature until use. Soil moisture was maintained at 70% capacity using sterile distilled water.
- Growth conditions: For the germination, incubate the Petri dishes with seeds in a growth chamber at 28 °C under a 13 h light/11 h dark photo period without any humidity control (PHCbi MLR-352, PHC Corp., Tokyo, Japan). Maintain a photosynthetic photon flux density (PPFD) of 50 μmol m^−2^ s^−1^. For the seedling cultivation, seedlings in a growth chamber were cultivated under a light cycle and temperature of 13 h light at 27 °C/11 h dark at 23 °C, PPFD 123 µmol m^−2^ s^−1^, and a relative humidity of 60% (FLI-2000A).

### 2.3. Chemical Solutions

The chemicals used in this study are as follows:

- Sodium hypochlorite (NaClO), used for surface sterilization of rice seeds;
- Ethanol, used for sterilization of hands, utensils, surfaces and for sterilizing metal textiles;
- 3-octanone (>98.0% (GC) purity, Tokyo Chemical Industry Co., Ltd., Tokyo, Japan);
- 3-octanol (>98.0% (GC) purity, Tokyo Chemical Industry Co., Ltd., Tokyo, Japan);
- 1-octen-3-one (>95.0% (GC) purity, Tokyo Chemical Industry Co., Ltd., Tokyo, Japan);
- 1-octen-3-ol (>98.0% (GC) purity, Tokyo Chemical Industry Co., Ltd., Tokyo, Japan).

### 2.4. Equipment

The list of equipment used during this study is as follows:

- Growth chamber for germination (PHCbi MLR-352, PHC Corp., Tokyo, Japan);
- Growth chamber for VCs exposure test (FLI-2000A; TOKYO RIKAKIKAI Co., Ltd., Tokyo, Japan);
- Alcohol lamp (Maruemu Corp., Osaka, Japan);
- Paper towel, Kim Towel (Nippon Paper Crecia Co., Ltd., Tokyo, Japan);
- Sterile disposable plastic Petri dishes (ϕ90 × 15 mm, Az One Corp., Tokyo, Japan);
- Autoclave (HICLAVE HVE-50, HIRAYAMA Manufacturing Corp., Tokyo, Japan);
- GA-7 Magenta boxes (77 × 77 × 97 mm; Merck KgaA, Darmstadt, Germany);
- LI-250A Light meter (LI-COR Environmental, Lincoln, NE, USA);
- Filter paper (MerkMillipore, Merck KgaA, Burlington, MA, USA, CAT No. RAWP02500);
- Polydimethylsiloxane/Divinylbenzene (PDMS/DVB) fiber assemblies of 65 μm (Pink/plain) (Supelco^®^, Merck KgaA, Darmstadt, Germany);
- Jeol JMS-T100GCV type GC-TOF Mass Spectrometer (JEOL Co., Ltd., Datum Solution Division MS Service Department, Tokyo Japan);
- Agilent 7890A GC System with FID, serial number CN14333069 (Agilent Technologies, Santa Clara, CA, USA);
- Plastic containers of 3 L (Sanada Seiko Co., Ltd., Osaka, Japan);
- High-density polyethylene (HDPE) film of 10 μm thickness (Fukusuke Kogyo Co., Ltd., Ehime, Japan);
- Polyvinyl chloride (PVC) film of 9 μm thickness (Pack Style, Co., Ltd., Aichi, Japan);
- A 18-8 Extra Thick Measuring Spoon 1/4 Spoon (Wadasuke Co., Ltd., Niigata, Japan);
- Clear glass vial (Supelco^®^) of 15 mL with a screw cap fitted with a PTFE/silicone septum (Merck KgaA, Darmstadt, Germany).
- Analytical balance (Sartorius Entris II BCE124i-1S, Merck KgaA, Darmstadt, Germany).
- Freeze dryer, (EYELA FD-5N, TOKYO RIKAKIKAI Co., Ltd., Tokyo, Japan)

## 3. Procedure

### 3.1. Setting of Non-Contact Exposure Methods for Mushroom FBs

#### 3.1.1. Preparation of Rice Seedlings

1. Sterilize seeds: Surface sterilize rice seeds using 1% (*w*/*v*) sodium hypochlorite for 15 min. Rinse the seeds five times with sterile distilled water.
2. Germinate seeds: Place sterilized seeds onto four-ply sterile Kim Towel moistened with 10 mL sterile distilled water in a Petri dish and sealed with surgical tape. Incubate the Petri dishes with seeds in a growth chamber for 4 days.

#### 3.1.2. Seedling Cultivation with Enokitake or Shiitake VCs Treatment

1. Transplant seedlings: Transfer five of the 4-day-old germinated rice seedlings into a Magenta box containing 200 g of sterile Kumiai synthetic culture soil with calcium silicate.

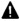

**CRITICAL STEP**: Only use germinated seeds of uniform germination stage. Handle seedlings gently to avoid root damage.

2. For the VCs treatment group (+VCs), place an open Petri dish containing 15 g of FBs in a 3 L plastic container alongside the seedlings that you sowed in the magenta box in step 1, above.
3. For the control group (−VCs), place seedlings in the filled Magenta box in identical containers without FBs.

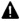

**CRITICAL STEP:** Ensure no direct contact between the FBs and seedlings to maintain non-contact VCs exposure.

4. Place containers in a growth chamber for 14 days.
5. Harvest and measure seedlings: After 14 days, harvest the seedlings and measure key growth parameters:
  - Plant height (cm): Measure from the base of the plant to the apex of the uppermost leaf.
  - Root length (cm): Measure from the apex of the longest root to the base of the plant.
  - Dry weight (mg): Freeze-dry seedlings for 24 h using an EYELA FD-5N freeze dryer and weigh using a Sartorius Entris II BCE124i-1S analytical balance.

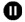

**PAUSE STEP:** Dried samples can be stored in desiccators for up to 1 week before weighing.

#### 3.1.3. Dose Optimization for Shiitake FBs

1. Germinate seeds: seed germination was performed as described in Section 3.1.1.
2. Set up dose treatments: Prepare six treatment groups by placing open Petri dishes containing varying quantities of shiitake FBs (0 g, 1 g, 5 g, 15 g, 30 g, and 60 g) in separate 3 L plastic containers. For the control group (0 g, −VCs), place seedlings in identical containers without FBs. And for the VCs treatment groups (+VCs), place Petri dishes with the specified quantities of FBs alongside the seedlings in each container.
3. Transplanting seedlings and seedling cultivation, harvest, and measurement were carried out as described in Section 3.1.2.

#### 3.1.4. Comparison of Fungi Bed Exposure Methods

1. Germinate seeds: seed germination was performed as described in Section 3.1.1.
2. Test exposure methods: Prepare 60 g of Shiitake FBs and evaluate three treatments:
  - HDPE Wrapping: Wrap FBs with 10 μm thick HDPE film (Fukusuke Kogyo Co., Ltd., Ehime, Japan).
  - PVC Wrapping: Wrap FBs with 9 μm thick PVC film (Pack Style Co., Ltd., Aichi, Japan).
  - Open Petri Dish (OPD): Leave FBs unwrapped in an open Petri dish (OPD).

Include a control group with no FBs.

3. Transplanting seedlings and seedling cultivation, harvest, and measurement were carried out as described in Section 3.1.2.

#### 3.1.5. Testing the Effects of Fungi Bed Aging

1. Seed germination was performed as described in Section 3.1.1.
2. Set up treatments: Divide seedlings into control (−VCs) and treatment (+VCs) groups. In the treatment group, expose seedlings to 60 g of shiitake FBs of different ages, categorized as YSFB, MSFB, and WSFB.
3. Transplanting seedlings and seedling cultivation, harvest, and measurement were carried out as described in Section 3.1.2.

### 3.2. Waste Shiitake Mushroom FBs Substractes-Derived VCs

#### 3.2.1. Volatile Compound Identification Via HS-SPME-GC-TOF-MS

1. Prepare fungi bed samples: Collect 2.5 g of WSFBs using a sterile knife. Place samples into 20 mL screw-cap vials and seal with silicone septa.
2. Extract volatiles: Heat vials in a 60 °C water bath for 10 min to dissipate the VCs. Then, insert SPME fiber assembly and extract volatiles. Incubate vials at 28 °C in a growth chamber for 24 h.
3. Analyze samples: Analyze extracted volatiles using a GC-TOF-MS with an Rtx-Wax capillary column. Use the parameters specified in Supplementary Table S1.

#### 3.2.2. Testing Synthetic Volatile Compounds

1. Germinate seeds: seed germination was performed as described in Section 3.1.1.
2. Prepare synthetic volatiles: Synthetic VCs exposure experiments were implemented using 3-octanone, 3-octanol, 1-octen-3-ol, and 1-octen-3-one, respectively. All synthetic VCs were solution type and analytical grade and used without further purification.
3. Mount the compound on filter paper: Test the compound at dose rates of 0.1 μL, 1 μL, 5 μL, and 10 μL. Drop the compounds onto the filter paper, place them on a Petri dish, and immediately place the Petri dish into 3 L containers.
4. Expose seedlings: Place Petri dishes inside 3 L containers housing rice seedlings in Magenta boxes.

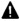

**CRITICAL STEP:** Resupply synthetic VCs on day 7 to maintain exposure.

5. Transplanting seedlings and seedling cultivation, harvest, and measurement were carried out as described in Section 3.1.2.

### 3.3. Workflow and Experimental Design

**Figure 1:**
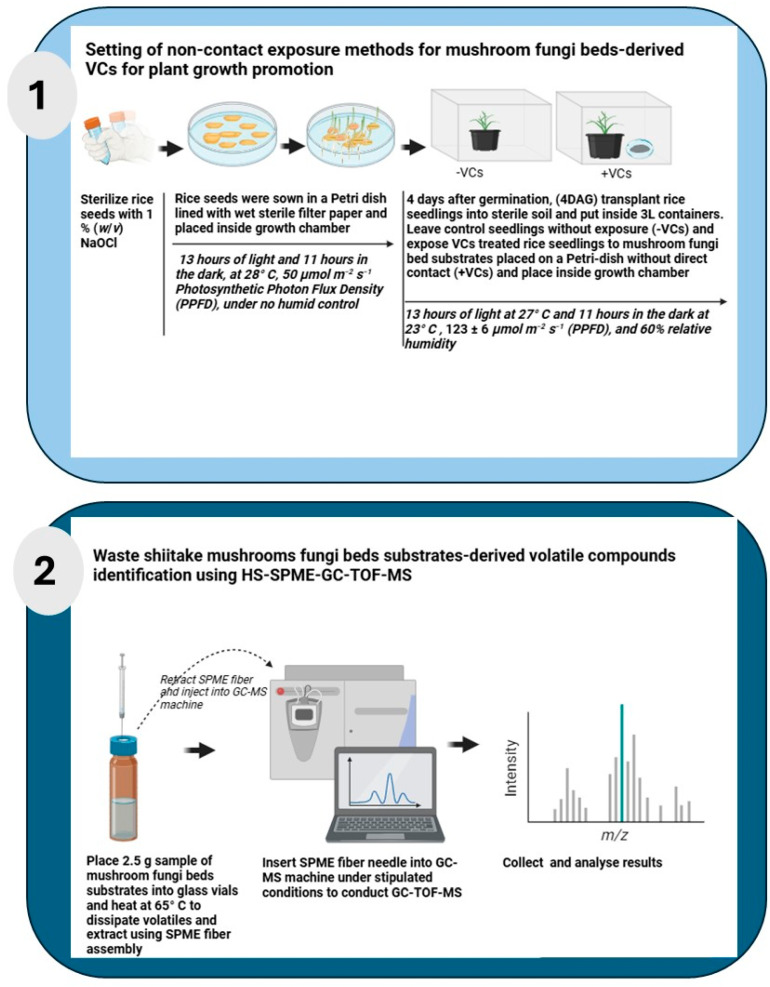
An overview of the schematic workflow of non-contact VCs exposure on rice seedlings experimental design and conditions. (**1**) Rice seeds were sterilized with 1% sodium hypochlorite. After 4 days of germination, rice seedlings were transplanted into a plant box with culture soil. Seedlings are exposed to mushroom fungi bed-derived VCs (+VCs) in a non-contact setup, while control seedlings remain unexposed (−VCs). Growth conditions include a 13 h light/11 h dark photoperiod, 123 ± 6 μmol m⁻^2^ s⁻^1^ and 122 ± 9 μmol m⁻^2^ s⁻^1^ PPFD, and around 60% relative humidity for 14 days. (**2**) HS-SPME-GC-TOF-MS analysis identified 3-octanone, 1-octen-3-ol, 1-octen-3-one, and 3-octanol as key fungal VCs present in shiitake mushroom FBs. Illustration was created using Biorender; https://BioRender.com (accessed on 3 March 2025).

The experiments were conducted using a simple two-point comparison method design to assess the effects of VCs emitted from mushroom FBs on rice seedling growth and biomass accumulation. Each treatment group consisted of five biological replicates (n = 5). The same experiment was carried out at least three times and representative data are presented. To minimize the influence of environmental fluctuations (slight differences from chamber to chamber or inside of chamber), the position of the 3 L container was changed within the growth chambers daily and every three days between the chambers. Figure 1 illustrates the experimental setup and analytical approach used to examine the effects of VCs derived from mushroom FBs on rice growth (1) and outlines the VCs identification process using Headspace Solid-Phase Microextraction Gas Chromatography Time-of-Flight Mass Spectrometry (HS-SPME-GC-TOF-MS) analysis (2).

**Section 1: Non-contact exposure for mushroom FBs-derived VCs for plant growth promotion:** Rice seeds were sterilized with 2% NaClO and germinated on wet sterile filter paper in Petri dishes. Then, these Petri dishes were placed inside a growth chamber and then germinated for 4 days under 13 h light/11 h dark at 28 °C, 50 μmol m^−2^ s^−1^ PPFD condition. Four days after germination (4DAG), five seedlings were transplanted into sterile culture soil within a Magenta box. Two Magenta boxes with seedlings were prepared, one used for the control group (−VCs), and another one used for VCs treatment group (+VCs). Both groups were transferred to 3 L containers (Supplementary Figure S1), which were then placed inside growth chambers and cultivated for 14 days. Two growth chambers were utilized where the containers for the control and the treatment group were rotated every day to ensure that variations in environmental conditions did not influence the experimental outcomes. Both growth chambers were maintained at 13 h of light at 27 °C and 11 h of darkness at 23 °C and relative humidity of around 60% growth conditions, while PPFD differed, with 123 ± 6 μmol m^−2^ s^−1^ in one growth chamber and 122 ± 9 μmol m^−2^ s^−1^ in the other one. Then, these seedlings were harvested and analyzed for growth and biomass indexes. The +VCs-treated group was compared to the -VCs control group to assess the effectiveness of VCs emitted by mushroom FBs in promoting plant growth and biomass production.

Various exposure conditions were tested:

- Types of edible mushroom for FBs—enokitake and shiitake FBs (15 g each);
- Dose optimization using different weight quantities of shiitake FBs—1 g, 5 g, 15 g, 30 g, and 60 g;
- Cover FBs with gas-permeable films;
- FBs maturity VCs tests—YSFB, MSFB, and WSFB;
- Synthetic VCs—compounds derived from waste shiitake FBs.

**Section 2: VCs identification using HS-SPME-GC-TOF-MS.** The sample preparation and extraction of VCs from waste shiitake FBs followed protocols described by Muto et al. (2023), with some modifications [33]. Briefly, 2.5 g sample of fresh waste shiitake FBs was clipped and placed into a 15 mL clear glass vial (Supelco^®^, Merck KGaA, Darmstadt, Germany) with a screw cap fitted with a PTFE/silicone septum (Sigma-Aldrich, St. Louis, MO, USA). The vials were heated at 65 °C for 10 min in a water bath to facilitate the release of VCs from the FBs. The VCs were captured on 65 μm Polydimethylsiloxane/Divinylbenzene (PDMS/DVB) SPME fiber assemblies (Supelco^®^, Merck KGaA, Darmstadt, Germany) for 24 h under dark conditions at 28 °C. Following extraction, the SPME fiber assembly was inserted directly into the GC injection port for analysis. The process adhered to the conditions outlined in Supplementary Table S1, which specify the column and other analytical parameters. The extracted VCs were analyzed using the GC-TOF-MS system (JMS-T100GCV GC-TOF Mass Spectrometer, JEOL Co., Ltd., Datum Solution Division MS Service Department, Tokyo, Japan). The system includes an Agilent 7890A GC System with FID (serial number: CN14333069) and an Agilent G4513A Injector (serial number: CN14260013).

### 3.4. Statistical Analysis

Data were analyzed using one-way ANOVA followed by Tukey’s multiple comparison test to assess significant differences among treatments (*p* < 0.05 was considered statistically significant). All data were organized and statistical analyses were conducted using Microsoft office excel 2026 (Microsoft Corporation, Redmond, WA, USA). Results are presented as mean ± standard deviation (SD), with significance levels indicated by different letters in figures and tables.

## 4. Results

### 4.1. Application of Method for Short-Term Seedling Cultivation with VCs from Edible Mushroom FBs Resulted in Significant Biomass Increase

To evaluate the growth effects of mushroom FBs-derived volatile compounds (VCs), rice seedlings were exposed to shiitake and enokitake FBs-derived VCs under controlled conditions, while control seedlings remained unexposed. After 14 days, plant height, root length, and biomass accumulation were measured.

**Figure 2:**
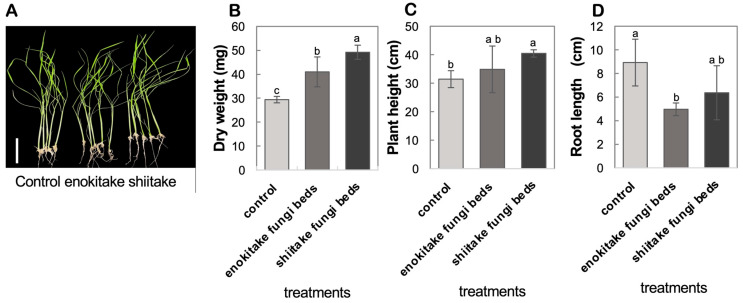
Effect of VCs emitted from enokitake and shiitake fungi beds on rice seedling growth and biomass accumulation. (**A**) Representative images of rice seedlings exposed to VCs from enokitake and shiitake fungi beds compared to control seedlings (scale bar = 5 cm is shown as white bar at the left). (**B**) Dry weight (mg) of rice seedlings after 14 days of exposure. (**C**) Plant height (cm) of rice seedlings. (**D**) Root length (cm) of rice seedlings. Distinct letters (a, b, c) denote statistically significant differences (*p* < 0.05) among the treatments. The data are presented as mean ± standard deviation (SD) with n = 5. Error bars represent the standard deviation (±SD).

The results showed distinct effects of VCs emitted from shiitake and enokitake FBs on rice seedling growth and biomass accumulation (Figure 2). Seedlings exposed to shiitake FBs VCs exhibited significantly highest dry weight, 49.2 ± 2.64 mg, representing a 67.4% increase compared to the control (29.4 ± 1.20 mg) (Figure 2B). Enokitake-exposed seedlings also showed an increase in dry weight (41.0 ± 5.59 mg), representing a 39.5% increase when compared with the control (Figure 2B). Similarly, plant height was significantly enhanced by shiitake VCs, whereby an average plant height of 40.4 ± 1.16 cm, a 28.7% higher than the control (31.4 ± 2.66 cm) was recorded (Figure 2C). Enokitake FBs VCs recorded a relatively lesser plant height of 34.9 ± 7.33 cm, leading to only a 10.9% increase, which was statistically non-significant (Figure 2C). Root development was inhibited in rice seedlings exposed to both shiitake and enokitake FBs VCs when compared to controls. Seedlings exposed to shiitake FBs VCs had slightly longer roots than those exposed to enokitake, averaging 6.4 ± 2.06 cm, a 30.4% reduction compared to the control (Figure 2D). A contrasting trend was observed in seedlings exposed to enokitake FBs which exhibited the most significant reduction in root length, averaging 5.0 ± 0.63 cm, a 45.6% decrease compared to the control (9.2 ± 1.83 cm) (Figure 2D). These results demonstrate that VCs from edible mushroom FBs are effective in promoting shoot growth and biomass accumulation. This suggests that the composition and activity of VCs may differ between FBs, influencing specific growth attributes in rice seedlings. In this study, shiitake FBs are shown to be a preferable source of VCs for plant growth and biomass promotion.

### 4.2. Results from Dose Optimization for Shiitake FBs

**Figure 3:**
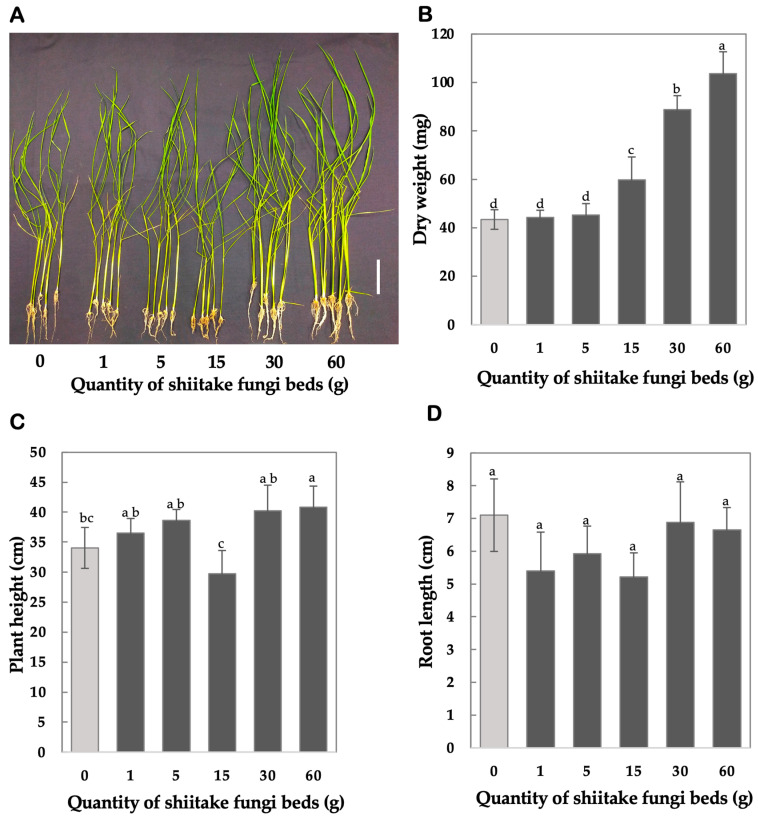
Dose–response effects of shiitake fungi bed VCs on rice seedling growth and biomass accumulation. (**A**) Representative images of rice seedlings after 14 days of non-contact exposure to varying quantities (0–60 g) of shiitake fungi bed substrates (scale bar = 5 cm is shown as white bar at the right). (**B**) Dry weight of rice seedlings. (**C**) Plant height. (**D**) Root length. Different letters (a, b, c, d) indicate statistically significant differences between treatments (Tukey’s test, *p* < 0.05). Data are presented as mean ± SD (n = 5). Error bars indicate the standard deviation (±SD).

To determine the optimal quantity of shiitake FBs for maximizing rice seedling growth and biomass accumulation, we evaluated the effects of different amounts of shiitake FBs on these growth parameters. The appearance of rice seedlings in Figure 3A shows the effect of varying quantities of shiitake FBs VCs on rice seedling growth and biomass production. The results demonstrate a clear dose-dependent effect of VCs emitted from shiitake FBs on rice seedling growth parameters, including dry weight, plant height, and root length. Dry weight significantly increased with 15, 30, 60 g of shiitake FBs. Seedlings exposed to 60 g had the highest dry weight (103.6 ± 9.07 mg, labeled a), representing a 138.7% increase compared to the control (43.4 ± 4.04 mg, labeled d, *p* < 0.0001). Similarly, seedlings exposed to 30 g showed a significant increase to 88.8 ± 5.76 mg (labeled b), a 104.6% increase relative to the control (*p* < 0.0001). At 15 g, dry weight was 59.8 ± 9.41 mg (labeled c) and significantly higher than the control (43.44 ± 4.04 mg) (*p* = 0.0062). In contrast, lower doses (1 g and 5 g) showed no significant differences compared to the control (44.2 ± 3.03 mg and 45.2 ± 4.76 mg, both labeled d, *p* > 0.05) (Figure 3B).

Plant height showed a dose-dependent response, with significant increases at 30 g and 60 g. Seedlings at 30 g and 60 g exhibited average plant heights of 40.2 ± 4.31 cm and 40.82 ± 3.54 cm, both labeled a, and significantly greater than the control (34.04 ± 3.40 cm, labeled b, c, *p* = 0.0305 and *p* = 0.0156, respectively). Lower doses, including 1 g, showed no significant difference from the control (36.5 ± 2.44 cm, labeled b, c, *p* > 0.05).

Root length exhibited a contrasting trend compared to dry weight and plant height, showing reductions across treatments. Seedlings exposed to 15 g had the shortest root lengths (5.22 ± 0.73 cm, labeled a, *p* = 0.0106), representing a significant 26.5% decrease compared to the control (7.1 ± 1.10 cm, labeled a). A significant reduction was also observed at 1 g (5.4 ± 1.18 cm, labeled a, *p* = 0.0255). For seedlings exposed to 5 g, root length averaged 5.92 ± 0.84 cm (labeled a), showing no statistical difference from the control (*p* > 0.05). However, at 30 g and 60 g, root lengths partially recovered to 6.88 ± 1.23 cm and 6.64 ± 0.69 cm, respectively, with no significant differences compared to the control (*p* > 0.05, labeled a) (Figure 3D).

These results suggest that increasing the quantity of FBs leads to a dose-dependent effect on rice seedling growth, with higher dose quantities potentially influencing biomass accumulation and development.

### 4.3. Comparison of Fungi Bed Exposure Methods

**Figure 4:**
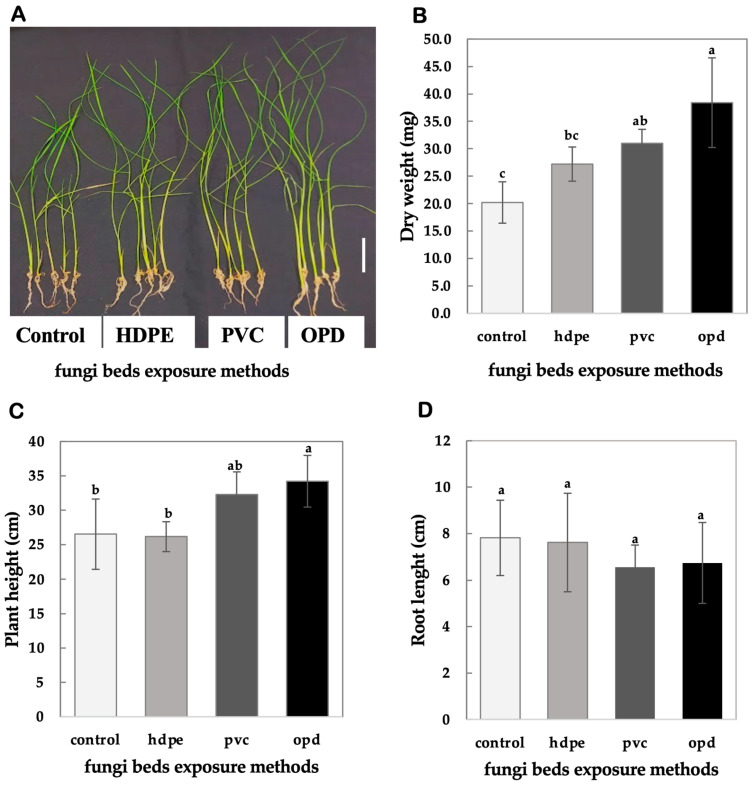
Effect of different exposure methods of shiitake fungi bed-derived VCs on rice seedling growth and biomass accumulation. (**A**) Representative image of rice seedlings exposed to shiitake fungi beds (60 g) using HDPE wrapping (HDPE), PVC wrapping (PVC), and open Petri dish (OPD) methods compared to the control (scale bar = 5 cm is shown as white bar at the right). (**B**) Dry weight (mg) of rice seedlings. (**C**) Plant height (cm) of rice seedlings. (**D**) Root length (cm) of rice seedlings. Different letters (a, b, c) indicate statistically significant differences among treatments (Tukey’s test, *p* < 0.05). Data are presented as mean ± SD (n = 5). Error bars indicate the standard deviation (±SD).

To investigate the effects of different exposure methods of shiitake FBs VCs, we encased FBs in gas-permeable polymer films. Rice seedlings were exposed to shiitake FBs (60 g) wrapped in gas-permeable polymer films of HDPE, PVC, or left unwrapped, and placed unwrapped in open Petri dishes (OPD). Figure 4A clearly shows the distinctive effects of different exposure methods of shiitake FBs (60 g) on rice seedling growth and biomass accumulation, favoring the OPD method.

The results demonstrate a clear variation in growth parameters, including dry weight, plant height, and root length, depending on the exposure method employed. Seedlings exposed to the OPD method showed the highest dry weight (38.4 ± 8.17 mg, labeled a, Figure 4B), which was significantly greater than the control (20.2 ± 3.77 mg, labeled c, *p* = 0.0019, Mean Diff. = −18.2, 95% CI: 8.01, 28.39) and the HDPE method (27.2 ± 3.11 mg, labeled b, c, *p* = 0.0126). The PVC method also significantly increased dry weight (31.0 ± 3.79 mg, labeled a, b, *p* = 0.0007, Mean Diff. = −10.8, 95% CI: [3.72, 17.88]), though to a lesser extent than OPD. Plant height followed a similar trend, with seedlings under the OPD method achieving the tallest average height (34.2 ± 3.75 cm, labeled a, Figure 4C), which was significantly higher than the control (26.5 ± 5.10 cm, labeled b, *p* = 0.0227, Mean Diff. = 7.7, 95% CI: [−0.13, 15.23]). The PVC method resulted in moderate increases in plant height (32.3 ± 3.28 cm, labeled a, b, *p* = 0.0664, Mean Diff. = 5.8, 95% CI: [−1.28, 12.88]), whereas the HDPE method (26.2 ± 2.63 cm, labeled b) showed no significant difference compared to the control (*p* = 0.8880, Mean Diff. = −0.3, 95% CI: [−6.74, 6.14]).

In contrast, root length exhibited no significant differences across treatments. The control group maintained an average root length of 7.8 ± 1.62 cm (labeled a, Figure 4D). Seedlings in the HDPE method (7.62 ± 2.12 cm, *p* = 0.8709, Mean Diff. = 0.20, 95% CI: [−3.08, 2.68]), PVC method (6.38 ± 1.07 cm, *p* = 0.6354, Mean Diff. = 1.26, 95% CI: [−0.49, 3.01]), and OPD method (6.74 ± 1.66 cm, *p* = 0.7355, Mean Diff. = 1.08, 95% CI: [−1.04, 3.20]) showed no significant deviations from the control, suggesting that root growth is less responsive to VCs exposure.

These findings clearly indicate that the OPD method is the most effective for enhancing rice seedling growth and biomass accumulation, with significant increases observed in both dry weight and plant height compared to other exposure methods. The PVC method exhibited moderate effects, while the HDPE method had the least impact, particularly on shoot growth and biomass. Root length remained unaffected across all treatments, emphasizing that shoot-related parameters are more responsive to VCs exposure under open and semi-permeable conditions (Figure 4).

### 4.4. Testing the Effects of Fungi Bed Aging

**Figure 5:**
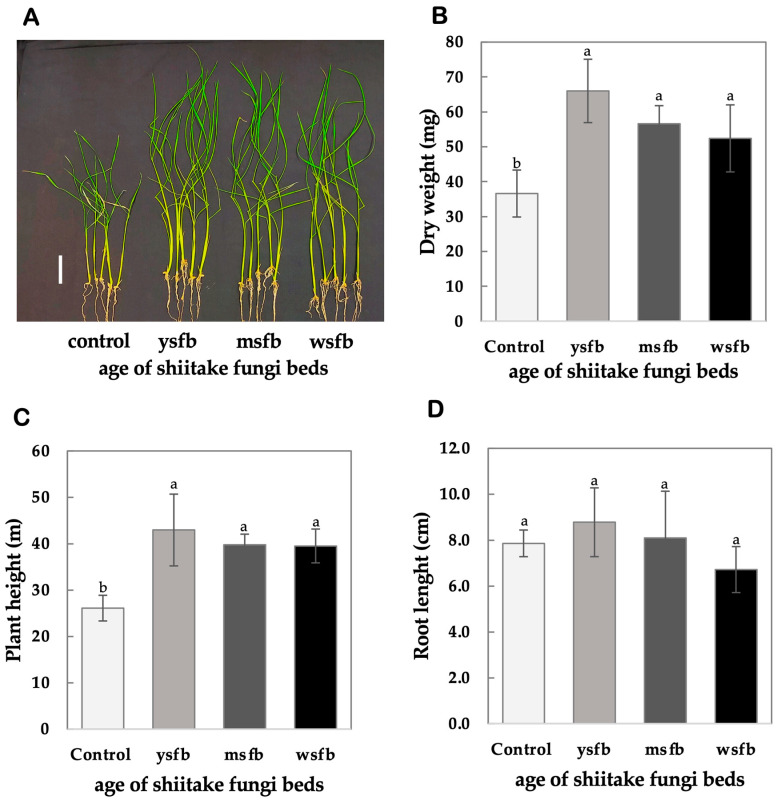
Effects of shiitake fungi bed age on rice seedling growth and biomass accumulation. (**A**) Representative images of rice seedlings exposed to volatile compounds (VCs) from young (YSFB), mature (MSFB), and waste (WSFB) shiitake fungi beds compared to the control (scale bar = 5 cm is shown as white bar at the left). (**B**) Dry weight (mg) of rice seedlings. (**C**) Plant height (cm) of rice seedlings. (**D**) Root length (cm) of rice seedlings. Different letters indicate statistically significant differences among treatments (Tukey’s test, *p* < 0.05). Data are presented as mean ± SD (n = 5). Error bars indicate the standard deviation (±SD).

The amount and type of VCs vary with the age of FBs, crucial for reusing waste FBs as biostimulants source. To assess the effects of different-aged shiitake FBs on rice seedling growth, 60 g of YSFB, MSFB, and WSFB were used. Dry weight, plant height, and root length were measured, as shown in Figure 5.

Dry weight (Figure 5B) showed the most significant improvements across treatments. Seedlings exposed to YSFB-VCs achieved the highest dry weight (41.2 ± 6.37 mg), with a significant mean difference of −29.40 mg compared to the control (21.8 ± 1.64 mg, *p* = 0.0001, 95% CI: −43.62 to −15.18, labeled b, c). Similarly, seedlings treated with MSFB-VCs exhibited a significant increase in dry weight (45.6 ± 9.52 mg), with a mean difference of −20.00 mg relative to the control (*p* = 0.0049, 95% CI: −34.22 to −5.78, labeled b, c). Exposure to WSFB-VCs also resulted in a significant increase (35.0 ± 7.57 mg) compared to the control, with a mean difference of −15.80 mg (*p* = 0.0268, 95% CI: −30.02 to −1.58, labeled b, c).

Plant height (Figure 5C) was significantly enhanced under YSFB-VCs and MSFB-VCs. Seedlings treated with YSFB-VCs displayed the greatest plant height (42.96 ± 7.72 cm) compared to the control (26.12 ± 2.77 cm) with a significant mean difference of −16.84 cm (*p* = 0.0002, 95% CI: −25.22 to −8.46, labeled a, b). Similarly, seedlings exposed to MSFB-VCs showed significant improvements in plant height (24.9 ± 3.34 cm) with a mean difference of −13.68 cm (*p* = 0.0013, 95% CI: −22.06 to −5.30, labeled a, b). Seedlings treated with WSFB-derived VCs exhibited a moderate increase in plant height (26.6 ± 3.10 cm), with a mean difference of −13.40 cm (*p* = 0.0016, 95% CI: −21.78 to −5.02, labeled a, b).

In contrast, root length (Figure 5D) did not show any significant differences across treatments. Tukey’s multiple comparisons test confirmed that seedlings treated with YSFB-VCs exhibited a mean root length of 8.74 ± 2.00 cm, which was not significantly different from the control (7.12 ± 2.00 cm, *p* = 0.7242, labeled a). Similarly, seedlings exposed to MSFB-VCs displayed a root length of 9.72 ± 1.89 cm (*p* = 0.9926, labeled a), while seedlings treated with WSFB-VCs showed a root length of 8.36 ± 1.05 cm, which also did not differ significantly from the control (*p* = 0.5764, labeled a).

Overall, results showed that YSFB and MSFB produced the most pronounced growth enhancement, significantly increasing dry weight and plant height (*p* < 0.05). However, WSFB also promoted moderate but significant growth, confirming that even after harvesting mushrooms, FBs retain bioactivity (Figure 5).

### 4.5. Results for Volatile Compound Identification Via HS-SPME-GC-TOF-MS

**Figure 6:**
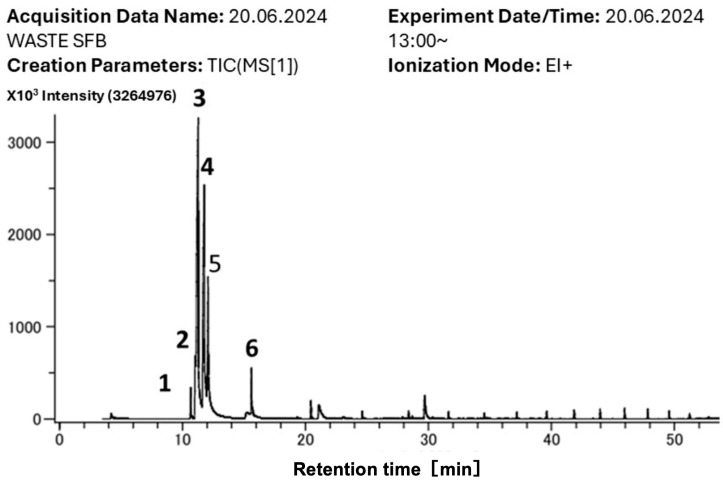
Representative GC-MS chromatogram of volatile compounds emitted from waste shiitake FBs (WSFB). The total ion chromatogram (TIC) shows six major peaks identified at different retention times, which correspond to distinct volatile organic compounds (VOCs). Peaks are labeled as follows: (1), (2), (3), (4), (5), and (6), indicating significant VOCs detected. Retention times and intensities of these peaks suggest the presence of dominant compounds contributing to the overall volatile emissions profile.

The GC-TOF-MS analysis of volatile compounds emitted from waste shiitake FBs identified six prominent compounds, which were categorized into ketones, alcohols, and siloxanes based on their molecular structure and chemical properties (Figure 6 and Table 1).

The results of HS-SPME-GC-TOF-MS analysis of VCs emitted from WSFB profiled six compounds, which were categorized into ketones, alcohols, and siloxanes based on their molecular structure and chemical properties (Table 1and Figure 6). Siloxanes may be coming from the coating materials of SPME fiber assembly (Table 1 and Figure 6, compound **1** and **6**).

Specific compounds, such as 1-Octen-3-one and 3-octanone (Table 1 and Figure 6, compounds **2** and **3**), have been directly linked to increased seedling biomass and plant height observed in earlier experiments [34,35]. 1-octen-3-ol (Table 1 and Figure 6, compounds **4**), commonly referred to as the “mushroom alcohol”, has been shown to trigger both systemic resistance and growth modulation in plants [36]. Similarly, 3-octanone (Table 1 and Figure 6) is recognized for its potential in agricultural applications, given its abundance and diversity of effects on plant metabolism, including inducing resistance to biotic and abiotic stresses [3,37].

### 4.6. Testing Synthetic Volatile Compounds

To test the effect of the identified VCs by HS-SPME-GC-TOF-MS on rice seedling growth, rice seedlings were cultivated in non-contact exposure manners on filter paper that was stained with various volume of the four key compounds: 1-Octen-3-one, 3-octanone, 1-octen-3-ol, and 3-octanol (Supplementary Figure S2). The optimal dose rates of synthetic VCs were initially determined based on the protocol described by Wood et al. (2022) [38], with minor modifications.

Rice seedlings were cultivated in Magenta boxes filled with sterile culture soil, ensuring no direct contact with the synthetic compounds. The organic compound solutions were administered at varying doses (0 μL [control], 0.1 μL, 1 μL, 5 μL, and 10 μL) by pipetting onto filter papers (1.2 μm pore size, type RA; Nihon Millipore Kogyo K.K., Tokyo, Japan) placed on Petri dishes. The cultivation setup was housed in 3 L covered containers, and the seedlings were grown under a 27 °C 13 h light/23 °C 11 h dark cycle condition for 14 days. The organic compound solutions were resupplied 7 days after treatment started.

**Figure 7:**
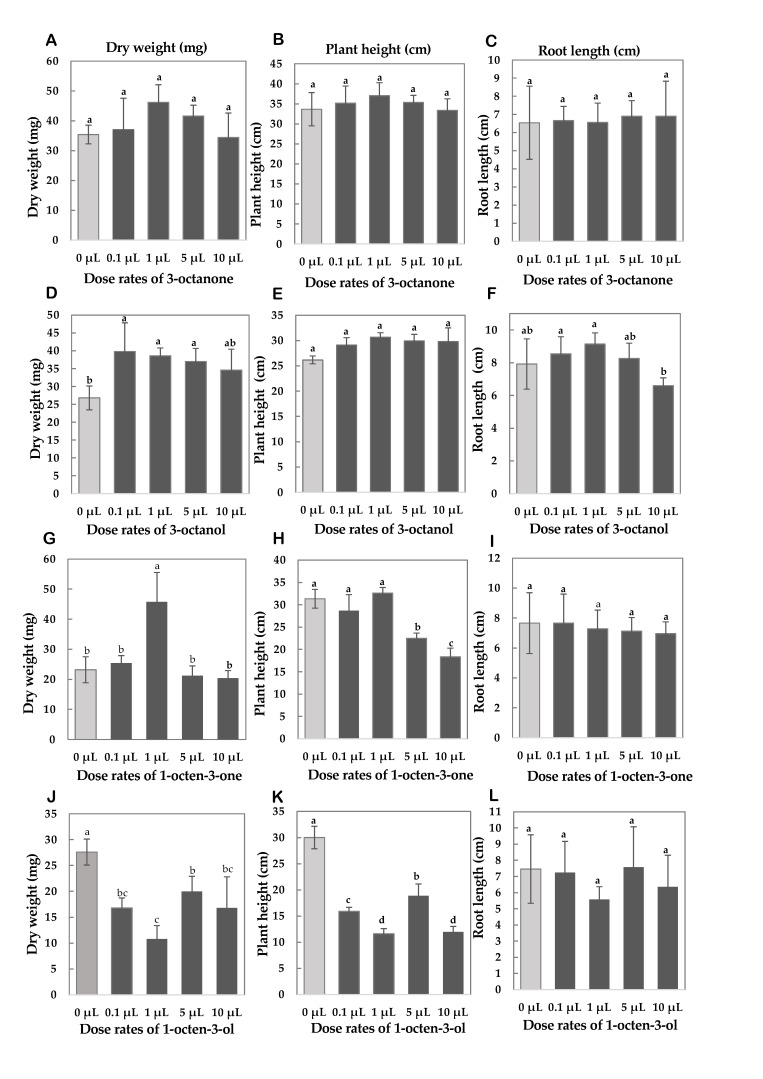
Growth responses of rice seedlings to increasing dose rates of 3-octanone, 3-octanol, 1-octen-3-one, and 1-octen-3-ol. (**A**–**C**) Dry weight, plant height, and root length under 3-octanone treatments. (**D**–**F**) Dry weight, plant height, and root length under 3-octanol treatments. (**G**–**I**) Dry weight, plant height, and root length under 1-Octen-3-one treatments. (**J**–**L**) Dry weight, plant height, and root length under 1-octen-3-ol treatments. Error bars indicate the standard deviation (mean ± SD). Letters above indicate significant differences between treatments (*p* < 0.05). The same letters indicate no statistical significance against the control while the different letters indicate statistical significance.

For 3-octanone, the dry weight peaked at 1 μL (46.2 ± 5.89 mg), reflecting a 30.5% increase compared to the control (35.4 ± 3.13 mg) (Figure 7A). The second highest dry weight occurred at 5 μL (41.6 ± 3.65 mg, +17.5%), while a decline was observed at 10 μL (34.4 ± 8.20 mg, −2.8%) (Figure 7A). The tallest plants were recorded at 1 μL (37.0 ± 3.25 cm, +10.1%) followed by 5 μL (35.3 ± 1.78 cm, +5.1%), whereas at 10 μL, the height decreased slightly (33.4 ± 2.90 cm, −0.8%) (Figure 7B). For root length, both 5 μL and 10 μL produced the longest roots (6.9 ± 0.86 cm and 6.9 ± 1.93 cm, +5.5%), with the control showing slightly shorter roots (6.5 ± 2.02 cm) (Figure 7C). However, despite these numerical differences, none of these changes were statistically significant (*p* > 0.05), indicating that 3-octanone did not produce a statistically confirmed effect on the measured variables at the evaluated doses.

For 3-octanol, the highest dry weight was observed at 0.1 μL (39.8 ± 8.04 mg, +48.5%), followed by 1 μL (38.6 ± 2.19 mg, +44.0%) (Figure 7D). At 10 μL, the dry weight declined to 34.6 ± 5.86 mg (+29.1%) (Figure 7D). Plant height was greatest at 1 μL (30.7 ± 0.88 cm, +17.1%), followed by 5 μL (29.9 ± 1.29 cm, +14.4%), but decreased at 10 μL (29.8 ± 2.71 cm, +13.8%) (Figure 7E). The longest root length was achieved at 1 μL (9.1 ± 0.68 cm, +15.4%), while the shortest occurred at 10 μL (6.6 ± 0.47 cm, −16.7%) compared to the control (7.9 ± 1.54 cm) (Figure 7F).

For 1-octen-3-one, the highest dry weight was at 1 μL (45.8 ± 9.76 mg, +97.4%), followed by 0.1 μL (25.4 ± 2.51 mg, +9.5%), while 10 μL resulted in the lowest dry weight (20.4 ± 2.51 mg, −12.1%) (Figure 7G). The tallest plants were recorded at 1 μL (32.6 ± 1.25 cm, +4.1%), with a sharp reduction at 10 μL (18.3 ± 1.96 cm, −41.5%) (Figure 7H). Root length showed minimal variation between the control and 0.1 μL (both at 7.7 cm), but the shortest roots were at 10 μL (7.0 ± 0.78 cm, −9.1%) (Figure 7I).

For 1-octen-3-ol, all treatments significantly inhibited growth and biomass (Figure 7J). The lowest dry weight was observed at 1 μL (10.8 ± 2.59 mg, −60.9%) compared to the control (27.6 ± 2.51 mg) (Figure 7I). Similarly, plant height was severely reduced at 1 μL (11.6 ± 0.99 cm, −61.3%) relative to the control (30.0 ± 2.15 cm) (Figure 7J). Root length was also shortest at 1 μL (5.6 ± 0.79 cm, −25.2%), while the control had the longest roots (7.5 ± 2.12 cm) (Figure 7K).

## 5. Discussion

Our study provides compelling evidence that VCs derived from edible mushroom FBs significantly enhance rice seedling growth and biomass accumulation. By utilizing a non-contact exposure method, we demonstrated that FBs-derived volatiles can act as effective plant growth-promoting biostimulants. These findings corroborate previous research on fungal volatile-mediated plant growth enhancement while addressing key challenges associated with their practical application on a large scale. The role of fungi-derived VCs in plant growth stimulation has gained increasing attention in recent years. Fungal volatiles modulate not only plant development but also stress tolerance and defense mechanisms through airborne signaling, eliminating the need for direct plant–fungi interactions [5,6,7]. Our study expands these findings by introducing edible mushroom FBs as a sustainable alternative to mycelial-based volatile production. Unlike conventional fungal cultivation systems, FBs offer a readily available, cost-effective, and scalable method for harnessing bioactive volatiles. Notably, our results confirm previous reports suggesting that waste FBs retain bioactive volatile activity even after mushroom harvesting [32,33]. Additionally, the wider agricultural benefits of FBs extend beyond VCs production. These materials have been explored for soil amendment, composting, and bioenergy applications, highlighting their broader sustainability potential [39,40]. By integrating waste mushroom FBs into circular agricultural practices, we can simultaneously enhance plant productivity and environmental sustainability.

*Application of mushroom FBs for short-term seedling cultivation*

Short-term exposure of rice seedlings to VCs from shiitake and enokitake FBs resulted in significant biomass increases. Results showed that shiitake FBs led to the highest dry weight and plant height and enokitake FBs provided a moderate increase in biomass but suppressed root elongation. This aligns with previous studies indicating that fungal VCs vary by species and can exert contrasting effects on plant morphology [26]. Moreover, previous studies have linked fungal volatile organic compounds (VOCs) to hormonal pathway modulation, nutrient uptake, and stress response regulation [3,31]. The ability of shiitake FBs to enhance seedling biomass suggests their potential as a natural alternative to synthetic growth enhancers.

*Dose-dependent effects of fungal volatiles on plant growth*

One of the most significant findings of this study was the dose-dependent response of rice seedlings to FBs-derived VCs. At 30 g and 60 g of shiitake FBs, results showed maximum biomass accumulation, while at 1 g and 5 g had negligible effects, and 15 g led to partial growth inhibition. This non-linear response aligns with previous research demonstrating that while low concentrations of fungal VCs promote growth, excessive exposure may trigger stress-related pathways leading to growth inhibition [8]. Similarly, Bitas et al. (2013) reported that high doses of 1-octen-3-ol could activate plant stress defense responses, ultimately suppressing growth [34]. Experiments using synthetic organic compounds further confirmed this phenomenon, whereby 1-Octen-3-one and 3-octanol promoted biomass accumulation at 1 µL but former inhibited growth at 10 µL. These results suggest that careful optimization of fungal VC concentrations is essential to maximize their beneficial effects.

*Optimization of non-contact exposure methods*

One of the primary objectives of this study was to develop and evaluate different non-contact exposure methods for fungal VCs. Our results indicate that the Open Petri Dish (OPD) exposure method yielded the highest biomass accumulation and plant height, while FBs wrapped in gas-permeable polymer films (PVC and HDPE) regulated volatile diffusion and influenced plant responses. These findings align with studies by Lee et al. (2015), which demonstrated that semi-permeable exposure systems optimize VC diffusion, enhancing plant responses [26]. Additionally, controlled release systems, including natural polymer-based formulations and volatile encapsulation technologies, may further enhance the targeted and sustained delivery of agrochemicals, improving efficiency and environmental sustainability in large-scale agricultural applications [41,42,43].

*Testing the effects of FBs aging*

Volatile emissions from young (YSFB), mature (MSFB), and waste (WSFB) shiitake FBs were analyzed for their biostimulatory effects. Results showed that YSFB and MSFB showed the strongest impact on dry weight and plant height. Notably, WSFB also promoted significant growth, confirming that FBs retain bioactivity even after mushroom harvesting. These findings suggest that waste shiitake FBs can be repurposed as an economical and sustainable alternative to chemical fertilizers. Moreover, environmental factors such as temperature, humidity, and aging stage may influence VC composition and activity, affecting their biostimulatory potential [26].

*Testing synthetic volatile compounds*

Our chemical analysis using HS-SPME-GC-TOF-MS identified 3-octanone, 3-octanol, 1-octen-3-one, and 1-octen-3-ol as predominant VCs. These findings align with previous studies that identified these VCs from mushroom [44]. Additionally, several studies have cited these compounds as having plant growth promoting and protection [36,45,46]. Our findings indicate that 1-octen-3-one and 3-octanol showed potential growth-promoting effects at lower dose rates, while higher dose rates were associated with reduced development or inhibition of growth. 1-octen-3-one enhanced biomass at 1 µL but was inhibitory at greater doses. In contrast, 1-octen-3-ol consistently suppressed plant growth across all tested concentrations. Our findings suggest that while 3-octanone did not show statistically significant differences in its different doses on dry weight, plant height, and root length, it may promote root proliferation at certain doses [47]. However, at higher concentrations, it might still have the potential to disrupt hormonal balance, possibly leading to growth inhibition. Meanwhile, 1-octen-3-ol remained inhibitory across all tested doses, aligning with previous research that highlights its role as an oxylipin messenger triggering stress-related responses, including the synthesis of methyl jasmonate, indole-3-acetic acid, and gibberellin according to a study by Estrada-Rivera et al. (2019) [48]. While these hormones are essential for plant development, excessive activation of defense mechanisms can shift resource allocation towards stress resistance at the expense of growth, which could explain our results [49]. The strong inhibitory effect of 1-octen-3-ol in our study further suggests that its role in plant–microbe interactions may be more oriented toward defense priming rather than direct growth promotion. Conversely, 3-octanol, which promoted rice seedling growth at moderate concentrations, is known to influence auxin/cytokinin homeostasis, stimulating lateral root formation while suppressing primary root elongation [50]. Additionally, it has been shown to enhance plant stress tolerance by modifying root architecture and supporting beneficial microbial interactions [38,51]. Our results indicate that while 3-octanol can be beneficial at appropriate doses, higher concentrations are likely to disrupt this balance, leading to adverse effects on seedling growth. These findings align with studies suggesting that fungal VCs influence plant hormonal balance and stress signaling [43,47]. The observed dose-dependent effects further confirm that microbial VCs act as signaling molecules, modulating plant physiology in a concentration-specific manner.

*Limitations and future research*

This study showed that VCs derived from mushroom FBs may promote plant growth. Commercial mushroom FBs may have different VCs and VCs amounts, when different companies are suppliers, because they use different substrate compositions and strains. Even in such cases, this protocol can provide a certain assessment of the effect on the plant. Future research could also address this by monitoring VC emissions qualitatively and quantitatively by GC-TOF-MS and other methods, as well as by monitoring gene expression and metabolic profiling on the plant side.

Additionally, the role of minor volatile compounds remains unclear. While key compounds like 1-octen-3-one and 3-octanol were identified using HS-SPME-GC-TOF-MS, secondary volatiles might not have been fully characterized. Advanced techniques such as GC×GC-TOF-MS and metabolic profiling could help determine their contributions. Improving VC delivery systems is also crucial.

## 6. Conclusions

This study highlights the potential of FBs-derived volatile compounds (VCs) as effective biostimulants for enhancing plant growth using non-contact exposure methods. Our findings demonstrate that 15 g of shiitake FBs VCs had a greater effect on promoting growth and biomass accumulation in rice seedlings compared to 15 g of enokitake FBs VCs. This is evidenced by a 67.4% (*p* < 0.05) increase in the dry weight of rice seedlings exposed to shiitake FBs VCs, while only a 39.5% (*p* > 0.05) increase was recorded in rice seedlings exposed to enokitake FBs VCs, compared to the control. Additionally, shiitake- FBs VCs promoted a 37.5% increase in plant height, whereas enokitake FBs VCs resulted in only an 18.5% increase, confirming their significant biostimulatory effects. Furthermore, the optimal doses of shiitake mushroom FBs for most pronounced growth promotion were observed at 30 g and 60 g, whereas lower doses (15 g) had minimal effects. Our results also showed that waste shiitake FBs promoted moderate but significant growth in rice seedlings, confirming that even post-harvest FBs retain bioactive volatile emissions. This validates the potential for repurposing waste mushroom FBs into agricultural biostimulants for plant growth. Experiments with synthetic volatiles revealed dose-dependent effects in 1-octen-3-one; low concentrations (1 μL) of 1-octen-3-one enhanced biomass, while higher concentrations of 1-octen-3-one suppressed growth. Interestingly, rice seedlings exposed to natural FBs VCs exhibited greater biomass accumulation and shoot elongation compared to those treated with individual synthetic volatiles. Overall, these findings underscore that natural FBs outperform synthetic individual volatiles in promoting plant growth, emphasizing the importance of whole volatile-emitting systems in sustainable agriculture. We conclude therefore that mushroom FBs show potential as a scalable, cost-effective, and environmentally friendly biostimulant technology that can be applied without direct contact, potentially reducing reliance on synthetic agricultural inputs.

## Figures and Tables

**Table 1:** Volatile compounds are detected in waste shiitake FBs by GC-TOF-MS analysis. Compounds are presented with retention times, molecular formulas, molecular weights, and chemical families.

Peak	RT (min)	Compound Name	Molecular Formula	Molecular Weight (g·mol^−1^)	Family
*1	10.2	Octamethylcyclotetrasiloxane	[(CH_3_)_2_SiO]_4_	296.62	Cyclomethicones
2	10.3	1-octen-3-one	C_8_H_14_O	126.20	Ketone
3	11.8	3-octanone	C_8_H_16_O	128.21	Ketone
4	12	1-octen-3-ol	C_8_H_16_O	128.21	Alcohol
5	12.1	3-octanol	C_8_H_18_O	130.23	Alcohol
*6	14.9	Decamethylcyclopentasiloxane	[(CH_3_)_2_SiO]_5_	370.77	Cyclomethicones

* 1 and 6 are probably from SPME fiber assembly coating material.

## Data Availability

Dataset available on request from the authors.

## Supplementary Materials

The following supporting information can be downloaded at: https://www.mdpi.com/article/10.3390/mps8020029/s1, Figure S1: Experimental container setup for non-contact exposure of rice seedlings to volatile compounds (VCs) emitted from mushroom FBs; Figure S2: Container setup for dose–response testing of synthetic individual volatile compounds (VCs) identified in waste shiitake FBs; Table S1: GC-TOF-MS conditions for analysis of VCs derived from waste shiitake FBs.
