# Supplementary material for: Development of an Application Method for Volatile Compounds Derived from Mushroom Fungi Beds as Plant Growth-Promoting Biostimulants
Source: Methods Protoc. 2025 Mar 7;8(2):29. doi: 10.3390/mps8020029 (PMC11932248; doi:10.3390/mps8020029)
Supplement: Supplementary file 1 [file mps-08-00029-s001.zip › mps-3481258-supplementary.pdf]

Table S1: GC-TOF-MS conditions for analysis of VCs derived from waste shiitake FBs.

Column	Type	Rtx-Wax capillary column
	Length (m)	60
	ID (mm)	0.25
	Df (μm)	0.25
Carrier Gas	Gas type	Helium (99.99% pure)
	Total flow	10 ml min-1
	Flow rate	1 ml min-1
	Purge flow	4 ml min-1
	Pressure	109.2 kPa
	Temperature	250 °C
Inlet	Injection mode	Splitless
Oven temperature program	Isothermal heating	5 min at 35 °C
	Temperature gradient	5 °C min-1 to 280 °C
	Final heating	15 min at 230 °C
Mass spectra	Mass scan method	Total-ion-count (TIC)
	Mass scan range	35 to 500 m/z
	Run time	1.5 to 59.0 min
	Event time	0.3 s

Supplementary Table S1 outlines the gas chromatography–time-of-flight mass spectrometry (GC-TOF-MS) conditions used to analyze VCs emitted from waste shiitake FBs. It details the chromatographic column specifications, carrier gas parameters, injection conditions, oven temperature program, and mass spectrometry settings.

- **Column Specifications**: The analysis was performed using an Rtx-Wax capillary column (60 m length, 0.25 mm internal diameter, 0.25 μm film thickness).
- **Carrier Gas Parameters**: High-purity helium (99.99%) served as the carrier gas, with a total flow rate of 10 mL/min, a column flow rate of 1 mL/min, and a purge flow of 4 mL/min at 109.2 kPa.
- **Injection Conditions**: The inlet temperature was set at 250 °C, and a splitless injection mode was used to enhance sensitivity.
- **Oven Temperature Program**: The program began with an isothermal hold at 35 °C for 5 minutes, followed by a temperature increase of 5 °C/min until reaching 280 °C. The final step maintained 230 °C for 15 minutes to ensure complete volatilization of compounds.
- **Mass Spectrometry Conditions**: Mass spectra were recorded using a total-ion-count (TIC) scan within a mass range of 35–500 m/z. The run time spanned 1.5 to 59.0 minutes, with an event time of 0.3 seconds.

These conditions were optimized to enable accurate and comprehensive profiling of volatile compounds, facilitating the identification of potential plant growth-promoting volatiles.

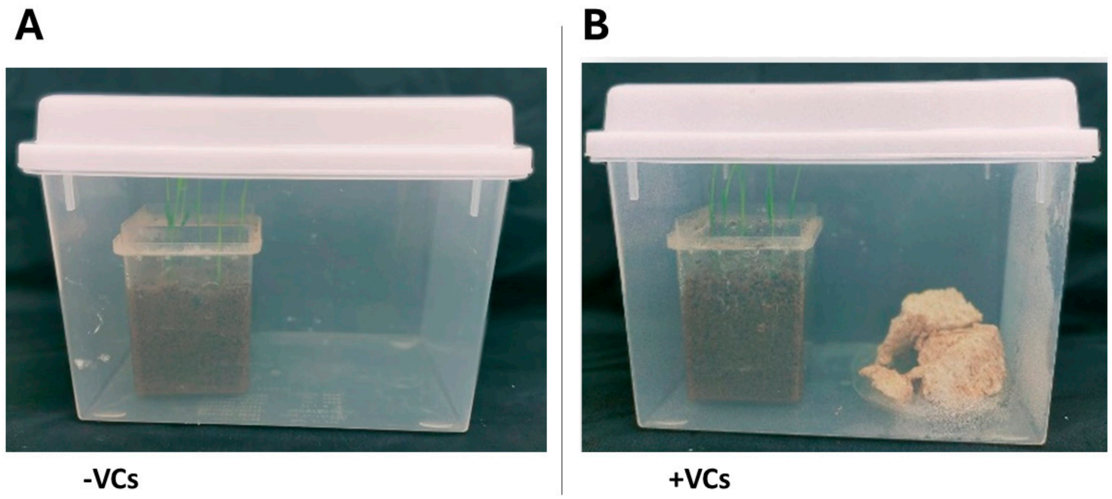

Figure S1: Experimental container setup for non-contact exposure of rice seedlings to volatile compounds (VCs) emitted from mushroom FBs. (A) Control treatment without volatile compounds (-VCs). (B) Treatment with volatile compounds (+VCs), where mushroom FBs substrates are placed on a Petri dish within the sealed container. The setup allows for the assessment of plant growth responses to emitted volatiles under controlled conditions.

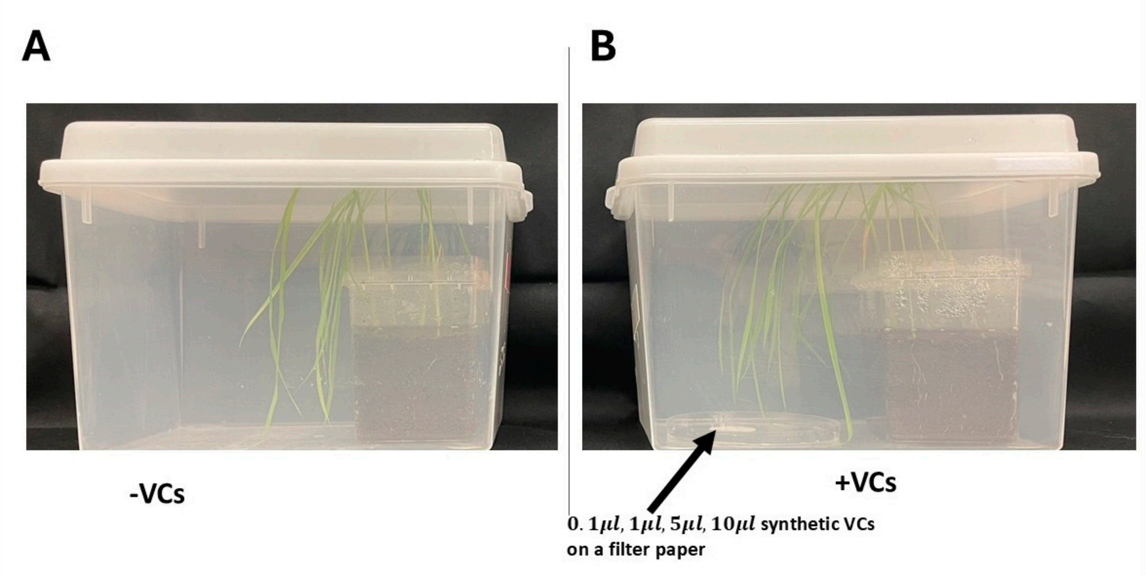

Figure S2: Container setup for dose–response testing of synthetic individual volatile compounds (VCs) identified in waste FBs. (A) Control setup without volatile compounds (-VCs). (B) Treatment setup with synthetic volatile compounds (+VCs) placed on a Petri dish within the sealed container. This experimental design allows for assessing the effects of specific VCs on rice seedling growth under controlled conditions.

To test the effect of the identified VCs by HS-SPME-GC-TOF-MS on rice seedling growth, rice seedlings were cultivated in non-contact exposure manners on filter paper that was stained with various volumes of the four key compounds: 1-Octen-3-one, 3-Octanone, 1-Octen-3-ol and 3-Octanol. The optimal dose rates of synthetic VCs were initially determined based on the protocol described by Wood et al. (2022), with minor modifications.

Rice seedlings were cultivated in Magenta boxes filled with sterile culture soil, ensuring no direct contact with the synthetic compounds. The organic compound solutions were administered at varying doses (0 μL [control], 0.1 μL, 1 μL, 5 μL, and 10 μL) by pipetting onto filter papers (1.2 μm pore size, type RA; Nihon Millipore Kogyo K.K., Tokyo, Japan) placed on Petri dishes. The cultivation setup was housed in 3L covered containers, and the seedlings were grown under a 27°C 13-hour light/ 23°C 11-hour dark cycle condition for 14 days. The organic compound solutions were resupplied 7 days after treatment started.
